# Human Leukocyte Antigen Genes and Interferon Beta Preparations Influence Risk of Developing Neutralizing Anti-Drug Antibodies in Multiple Sclerosis

**DOI:** 10.1371/journal.pone.0090479

**Published:** 2014-03-07

**Authors:** Jenny Link, Malin Lundkvist Ryner, Katharina Fink, Christina Hermanrud, Izaura Lima, Boel Brynedal, Ingrid Kockum, Jan Hillert, Anna Fogdell-Hahn

**Affiliations:** 1 Department of Clinical Neuroscience, Karolinska Institutet, Stockholm, Sweden; 2 Institute of Environmental Medicine, Karolinska Institutet, Stockholm, Sweden; University of Düsseldorf, Germany

## Abstract

A significant proportion of patients with multiple sclerosis who receive interferon beta (IFNβ) therapy develop neutralizing antibodies (NAbs) that reduce drug efficacy. To investigate if HLA class I and II alleles are associated with development of NAbs against IFNβ we analyzed whether NAb status and development of NAb titers high enough to be biologically relevant (>150 tenfold reduction units/ml) correlated with the HLA allele group carriage in a cohort of 903 Swedish patients with multiple sclerosis treated with either intramuscular IFNβ-1a, subcutaneous IFNβ-1a or subcutaneous IFNβ-1b. Carriage of HLA-DRB1*15 was associated with increased risk of developing NAbs and high NAb titers. After stratification based on type of IFNβ preparation, HLA-DRB1*15 carriage was observed to increase the risk of developing NAbs as well as high NAb titers against both subcutaneous and intramuscular IFNβ-1a. Furthermore, in patients receiving subcutaneous IFNβ-1a carriage of HLA-DQA1*05 decreased the risk for high NAb titers. In IFNβ-1b treated patients, HLA-DRB1*04 increased the risk of developing high NAb titers, and in a subgroup analysis of DRB1*04 alleles the risk for NAbs was increased in DRB1*04:01 carriers. In conclusion, there is a preparation-specific genetically determined risk to develop NAbs against IFNβ high enough to be clinically relevant in treatment decisions for patients with multiple sclerosis if confirmed in future studies. However, choice of IFNβ preparation still remains the single most significant determinant for the risk of developing NAbs.

## Introduction

Recombinant interferon beta (IFNβ) is a first-line therapy for relapsing-remitting multiple sclerosis (MS) and its therapeutic effect includes reduced frequency of clinical relapses and new lesions [1], [2] and reduced accumulation of disability over time [3]. Two types of recombinant IFNβ products are used for treatment of MS, IFNβ-1a and IFNβ-1b. The IFNβ-1a preparations are injected either at 30 µg intramuscularly (i.m.) once a week or at 22 µg and 44 µg subcutaneously (s.c.) three times a week, and the IFNβ-1b preparation is injected at 250 µg s.c. every other day. IFNβ-1a is identical in amino acid sequence to human IFNβ, while IFNβ-1b lacks the N-terminal methionine (Met_1_) and the asparagine-linked glycosylation at position 80 (Asn_80_) and has a cysteine-to-serine substitution at position 17 (Cys_17_Ser).

A proportion of MS patients receiving IFNβ treatment develop neutralizing anti-drug antibodies (NAbs), which at high titers block the biological response [4], [5] and the therapeutic efficacy [6]–[8] of IFNβ. The frequency of NAb positive patients differs depending on the IFNβ preparations used with IFNβ-1b inducing NAbs in 28%–47% of treated patients, s.c. IFNβ-1a in 5%–28% and i.m. IFNβ-1a in 2%–6% [9]. Among the NAb positive patients, the proportion with high NAb titers has been reported to be 19%–73% if treated with IFNβ-1b, 52%–79% if treated with s.c. IFNβ-1a and 19%–79% if treated with i.m. IFNβ-1a [10]–[15].

Differences in immunogenicity between preparations are likely due to factors such as dosage, injection frequency, application site, glycosylation and protein aggregate formation, and particle contents [9], [16], [17]. Since not all IFNβ-treated patients develop NAbs, other patient-specific immunological factors seem to influence this risk. The human leukocyte antigen (HLA) region on chromosome 6p21 carries the genes with the strongest effect on MS susceptibility and has also been linked to the immunogenicity of IFNβ therapy. An association was shown between the DRB1*15:01-DQB1*06:02 haplotype and increased proliferative response of CD4+ T-cells to specific IFNβ epitopes *in vitro* [18]. Furthermore, a study on peripheral blood mononuclear cells from IFNβ-treated MS patients found an association between the HLA-DRB1*07:01-DQA1*02:01 haplotype and IFNβ immunogenicity [19]. Recent studies based on larger patient materials have associated an increased risk of developing anti-drug antibodies against IFNβ with the HLA-DRB1*04:01 and HLA-DRB1*04:08 alleles [20], [21]. Also, genetic associations of an intergenic single nucleotide polymorphism (SNP) on chromosome 8q24.3 (rs4961252) and a SNP within the HLA region (rs9272105), independent of DRB1*04:01 and DRB1*04:08, with the development of high anti-drug antibody titers against IFNβ have been reported [22].

The aim of this study was to investigate if HLA class I and II allele groups are associated with risk of developing NAbs in a Swedish cohort of IFNβ-treated MS patients and if there is an HLA influence on biologically relevant high NAb titers.

## Materials and Methods

### Ethics statement

The study obtained approval from the ethical board of Karolinska Institutet, Stockholm, Sweden, and subsequently genotyped blood samples were obtained only from patients who gave their oral and written informed consent in accordance with the ethical permissions.

### Patients included in the study

In June 2012, the Swedish NAb biobank of routine serum samples contained more than 7000 samples from more than 4000 unique patients. Cross-referencing with the genetic database resulted in 1726 patients with both NAb status and HLA genotype data, either by classical typing (*HLA-A*, *HLA-C* and *HLA-DRB1* by Olerup SSP™ HLA Low resolution Kit [23] and *HLA-B* by Luminex based reverse SSO Labtype® SSO from One Lambda Inc., Canoga Park, CA, USA) or imputed either by using HLA*IMP:01 [24] with genotypes from the IMSGC WTCCC2 MS genome wide association study (GWAS) [25], or with HLA*IMP:02 [26] using genotypes from the Immunochip [27]. The threshold of the quality score Q for using imputed genotypes was 0.7. In cases where several sources of a HLA genotype were found, classical genotyping data was primarily used, and for imputed data Immunochip was predominantly used whereas GWAS derived data was only occasionally used. Comparison of genotypes between these sources yielded 1068 pairs of which 952 (89.1%) were concordant for both alleles, whereas 68 (6.4%) were discordant for one or both alleles. For 48 of the 1068 (4.5%) pairs, one allele was concordant but the other allele remained unknown from imputation due to the quality cut off. Genotypes for *HLA-DQA1* and *HLA-DQB1* were derived only from imputation of SNP data (Table S1). Classically subtyped genotypes and imputed genotypes from GWAS and Immunochip SNP data were used to identify HLA-DRB1*04:01 and HLA-DRB1*04:04 positive patients. NAb positive patients were defined as those ever having a positive sample, and patients ever having a NAb titer above 150 tenfold reduction units per milliliter (TRU/ml) were classified as high positive. NAb negative patients were required to never being tested positive and having at least one negative sample taken after twelve months of treatment. After exclusion of patients of non-Scandinavian origin (n = 403), defined as self-reported or by principal component analysis (PCA) performed within other projects [25], [27]and patients not fulfilling the criteria for NAb positivity and negativity (n = 420), 364 NAb positive and 539 NAb negative patients were included in the study. Information on the type of IFNβ preparation used was available for 358 of the 364 NAb positive patients and for all NAb negative patients (Table 1).

**Table 1:** Characteristics of the cohort.

	Total	NAb positive a	NAb negative
**Number**	903	364	539
**Age (mean years, IQR)**	48.4 (39–57)	51.2 (43–58)	46.5 (38–55)
**Female (%)**	69.9	65.9	72.5
**Titers >150 TRU/ml**	209	209	0
**IFNβ-1a i.m.**	346	49	297
**IFNβ-1a s.c.**	355	172	183
**IFNβ-1b**	196	137	59

a IFNβ preparation could be assigned to 358 of the 364 NAb positive patients, while the type of preparation used was unspecified for 6 NAb positive patients.

Abbreviations: IQR = interquartile range, i.m. = intramuscular, s.c. = subcutaneous, TRU/ml = tenfold reduction units per milliliter, IFNβ = interferon beta, NAb = neutralizing antibodies

### Analysis for neutralizing anti-drug antibodies

The antibody neutralizing activity in serum was assessed at the NAb laboratory at Karolinska Institutet in Stockholm. Patient samples sent to the laboratory in 2003–2006 were analyzed with the myxovirus resistance protein A (MxA) assay (MPA) [15] while later samples were analyzed with the MxA gene expression assay (MGA) as previously described [14], [28]. A strong correlation of NAb titers was found when 118 serum samples were titrated with the MPA and the MGA assay in our laboratory, and the methods can thus be considered to yield similar results (Spearman r = 0.93, 95% confidence interval 0.90 to 0.95, p value<0.0001). In both assays, titers were adjusted using the Kawade formula [29] in Softmax Pro (Softmax Pro Software, Sunnyvale, CA) and expressed as TRU/ml. Patients' NAb status was classified according to the following titer categories: negative (<10 TRU/ml), positive (>10 TRU/ml) and high positive (>150 TRU/ml). High NAb titers >150 TRU/ml markedly affect the *in vivo* bioactivity of IFNβ therapy by reducing the MxA gene expression in treated patients [5]. Titers >150 TRU/ml were therefore considered as biologically relevant titers.

### Treatment groups

Treatment information was obtained from the Swedish Multiple Sclerosis registry (SMS-reg; http://www.msreg.net) with permission from the board. Patients were categorized based on the IFNβ preparation (i.m. IFNβ-1a, s.c. IFNβ-1a or IFNβ-1b) used at the time of defined NAb status. NAb positive patients and those with biologically relevant titers were categorized based on the preparation used at the sampling date of the first positive sample and the first sample with a titer >150 TRU/ml, respectively.

### Allele frequency and carriage of alleles

The frequencies of all analyzed HLA alleles are presented in Table S2 and S3. Within each treatment group, all allele groups with a count of at least ten carriers in the positive and negative group were analyzed for association with development of NAb as well as with biologically relevant titers.

### Analysis of HLA-DRB1*04:01 and HLA-DRB1*04:04

Since not all DRB1*04 positive patients were subtyped to 4-digit resolution, the association analysis of specific DRB1*04 alleles to NAb development had to be weighted to compensate for this lack of information. A univariate association analysis was done by comparing carriers of a certain DRB1*04 allele against the DRB1*04 negatives together with DRB1*04 positives who were subtyped but who did not carry the allele in question. Hence, DRB1*04 positive individuals who were not subtyped were excluded from analysis. Since high resolution data were available only for a subset (88.4%) of the DRB1*04 positive patients and the proportion of subtyped patients was uneven between the NAb positive and NAb negative group (Table S6), this difference influences the result of a normal association analysis. To overcome this problem the reference population (DRB1*04 negative) was weighted when calculating the association of DRB1*04 alleles. This weighing constant is based on the percentage of subtyped individuals within the NAb positive and NAb negative groups and keeps the proportions of DRB1*04 positive intact through the analysis. For example, for the NAb positive individuals, 105 out of 120 DRB1*04 positive individuals were subtyped (87.5%), and this group was thus compared with 87.5% of the number of DRB1*04 negatives (in this case 207.4 of 237).

DRB1*04:08 could not be imputed due to low frequency in the reference panel used by HLA-IMP:01/IMP:02. The Swedish genetic material from the Epidemiologic Investigation of Rheumatoid Arthritis (EIRA) study [30], which also has been genotyped with the Immunochip and imputed with HLA*IMP:02 and have classical DRB1*04 subtyping data, was used as a cohort for checking HLA imputation accuracy. We found that 33 out of 36 known DRB1*04:08 positive individuals were imputed as DRB1*04:01 and three individuals were imputed as DRB1*04:04. Since both DRB1*04:01 and DRB1*04:08 have been identified as risk alleles for NAb development [21] it was decided to use and analyze the imputed data as it was, even though the estimate of DRB1*04:01 will be too high and DRB1*04:08 too low. Among the 68 classically genotyped patients there was only one patient carrying DRB1*04:08 (1.5%). Of the 185 patients with imputed high resolution data for DRB1*04 we estimate that two or three patients (based on the percentage found in classical typing) are assigned DRB1*04:01 instead. Since we know that the frequency of DRB1*04:08 is incorrectly estimated due to the problems with imputation, and the frequency obtained from classical genotyping was low, we decided not to analyze an association to DRB1*04:08 in detail. The single classically typed DRB1*04:08 positive patient was NAb negative and received s.c. IFNβ-1a.

### Statistics

All calculations were done in R version 2.15.1 [31]. Fisher's exact test (fisher.test in R) was used for all comparisons; two-tailed *P*-values were reported and considered significant if below alpha 0.05. The Bonferroni method was used to correct *P*-values for multiple comparisons (*P_C_*).

### Treatment specific absolute risk of NAb development

The absolute risk (AR) for development of NAbs and biologically relevant titers was used to assess the influence of HLA allele group carriage within each of the three treatment groups. The AR was estimated using Bayes' theorem [32]:
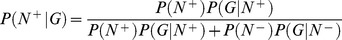
where G was the allele group carriage and N^+^ and N^−^ represented NAb development or negativity (or presence of biologically relevant titers and negativity). P(N^+^) was the frequency of NAb development or biologically relevant titers within each treatment group regardless of genotype.

### HLA binding prediction to IFNβ

The NetMHCII and NetMHCpan prediction servers [33], [34] were used for binding prediction of recombinant IFNβ-1a (166 amino acids) and recombinant IFNβ-1b (165 amino acids) to HLA class II alleles DRB1*04:01, DRB1*04:04 and DRB1*15:01, and the HLA class I alleles B*07:01 and C*07:01. The IFNβ sequences were obtained from DrugBank (www.drugbank.ca; Accession numbers: DB00060 and DB00068).

## Results

### Association of HLA alleles with NAb development

An association to DRB1*15 was observed for both development of NAbs (OR 1.43; *P_c_* = 0.036) and biologically relevant NAb titers (OR 1.58; *P_c_* = 0.01). Carriage of HLA-B*07 was associated with development of biologically relevant titers (OR 1.61; *P_c_* = 0.0497) (Table 2). Because of the high linkage disequilibrium (LD) within the HLA, a stratification on carriage of DRB1*15 was performed to assess whether B*07 was independently associated to the development of biologically relevant titers. Since B*07 did not show an independent association within either stratum we assume that this association is due to LD with DRB1*15. All analyzed HLA allele groups can be found in Table S2 and S3.

**Table 2:** HLA association to development of NAbs and biologically relevant titers.

	NAb development						Biologically relevant titers					
HLA allele a	No. NAb positive (%)	No. NAb negative (%)	Total cohort (%)	OR (95% C.I.)	P b	P_C_ c	No. BRT positive (%)	No. NAb negative (%)	Total cohort (%)	OR (95% C.I.)	P b	P_C_ c
A*02	206 (28.9)	248 (24.0)	26.0	1.28 (1.03–1.59)	0.026	1	119 (28.9)	248 (24.0)	25.4	1.28 (0.99–1.66)	0.061	1
B*07	185 (28.5)	203 (22.2)	24.8	1.4 (1.11–1.76)	0.0052	0.40	118 (31.4)	203 (22.2)	24.8	1.61 (1.23–2.10)	<0.001	0.0497
B*35	23 (3.6)	60 (6.7)	5.4	0.52 (0.32–0.85)	0.0084	0.64	11 (3.0)	60 (6.7)	5.6	0.43 (0.22–0.83)	0.010	0.76
C*07	279 (42.4)	352 (38.0)	39.8	1.2 (0.98–1.47)	0.086	1	175 (46.5)	352 (38.0)	40.5	1.42 (1.11–1.81)	0.015	1
DQA1*05	61 (11.9)	122 (17.9)	15.3	0.62 (0.44–0.86)	0.0045	0.34	28 (9.5)	122 (17.9)	15.4	0.48 (0.31–0.75)	<0.001	0.052
DQB1*05	31 (5.8)	76 (11.2)	8.8	0.49 (0.32–0.75)	0.0010	0.079	17 (5.5)	76 (11.2)	9.4	0.46 (0.27–0.79)	0.0046	0.35
DQB1*06	269 (50.2)	304 (44.7)	47.1	1.25 (0.99–1.56)	0.064	1	160 (51.6)	304 (44.7)	46.9	1.32 (1.01–1.73)	0.047	1
DRB1*01	34 (4.8)	85 (8.1)	6.8	0.58 (0.38–0.87)	0.0087	0.66	20 (4.9)	85 (8.1)	7.2	0.59 (0.36–0.97)	0.041	1
DRB1*03	56 (8.0)	124 (11.9)	10.3	0.65 (0.46–0.90)	0.010	0.77	32 (7.9)	124 (11.9)	10.8	0.64 (0.43–0.96)	0.030	1
DRB1*04	124 (17.4)	171 (16.3)	16.8	1.08 (0.83–1.39)	0.60	1	79 (19.2)	171 (16.3)	17.1	1.21 (0.9–1.63)	0.22	1
DRB1*15	291 (41.6)	348 (33.3)	36.6	1.43 (1.17–1.74)	<0.001	0.036	178 (44.1)	348 (33.3)	36.3	1.58 (1.25–2.00)	<0.001	0.012

a Major HLA alleles (>5% allele frequency) nominally associated or previously reported to be associated with development of NAbs and biologically relevant titers are presented. All HLA alleles can be found in **Table S2** and **S3**.

b nominal *P*-values assessed by Chi square test,

c Bonferroni corrected *P*-values (76 allele groups tested).

Abbreviations: BRT = biologically relevant titers, C.I. = confidence interval, NAb = neutralizing antibodies, OR = odds ratio.

### Treatment-dependent NAb development

In agreement with previous studies [9], [11], [15], [35], the frequency of NAbs was highest for IFNβ-1b and lowest for i.m. IFNβ-1a, whereas high titers were most frequently induced by s.c. IFNβ-1a, regardless of allele carriage. In the Swedish NAb registry the frequency of NAb development, independent of HLA allele group carriage, was 9.0% for i.m. IFNβ-1a, 33.6% for s.c. IFNβ-1a and 49.3% for IFNβ-1b. The frequency of biologically relevant titers was 3.9% for i.m. IFNβ-1a, 19.0% for s.c. IFNβ-1a and 15.9% for IFNβ-1b (Table S4).

### HLA allele associations in the three different treatment groups

Since NAb development is influenced by the IFNβ preparation we analyzed association of HLA alleles within strata based on the IFNβ preparation used. The AR of NAb development was also calculated for each allele group to assess how HLA allele group carriage influenced the risk of developing NAbs.

After correction for multiple testing DRB1*15 carriage was associated with biologically relevant titers (OR 4.36; *P_c_* = 0.044; AR 5.8%) for patients receiving i.m. IFNβ-1a (Table 3). All analyzed HLA allele groups can be found in Table S5.

**Table 3:** Carrier frequency and absolute risk for HLA allele groups associated to NAb development and biologically relevant titers^a^.

	NAb development						Biologically relevant titers					
i.m. IFNβ-1a	No. NAb positive (%)	No. NAb-negative (%)	OR (95% C.I.)	P^d^	P_C_ e	AR (%) c	No. BRT positive (%)	No. NAb negative (%)	OR (95% C.I.)	P^d^	P_C_ e	AR (%) c
**Treatment only** b	n/a	n/a	n/a	n/a	n/a	9.0	n/a	n/a	n/a	n/a	n/a	3.9
DQB1*06	31 (93.9)	141 (71.9)	6.05 (1.4–26.13)	0.0046	0.055	11.4	17 (94.4)	141 (71.9)	6.63 (0.86–51.04)	0.047	0.57	5.1
DRB1*15	34 (72.3)	159 (55.8)	2.07 (1.05–4.09)	0.038	0.46	11.3	22 (84.6)	159 (55.8)	4.36 (1.46–12.97)	0.0036	0.044	5.8

	NAb development						Biologically relevant titers					
s.c. IFNβ-1a	No. NAb positive (%)	No. NAb negative (%)	OR (95% C.I.)	P^d^	P_C_ e	AR (%)^c^	No. BRT positive (%)	No. NAb negative (%)	OR (95% C.I.)	P^d^	P_C_ e	AR (%)^c^
**Treatment only** b	n/a	n/a	n/a	n/a	n/a	33.6	n/a	n/a	n/a	n/a	n/a	19.0
A*03	68 (39.8)	57 (32.6)	1.37 (0.88–2.12)	0.18	1	38.2	50 (44.2)	57 (32.6)	1.64 (1.01–2.68)	0.047	1	24.1
B*07	86 (54.8)	56 (37.1)	2.06 (1.3–3.24)	0.0021	0.08	42.8	66 (64.7)	56 (37.1)	3.11 (1.84–5.25)	<0.001	<0.001	29.0
C*07	112 (70.9)	91 (59.9)	1.63 (1.02–2.62)	0.043	1	37.5	81 (78.6)	91 (59.9)	2.47 (1.39–4.37)	0.0017	0.066	23.5
DQA1*01	107 (90.7)	89 (82.4)	2.08 (0.94–4.59)	0.079	1	35.8	74 (94.9)	89 (82.4)	3.95 (1.29–12.12)	0.012	0.47	21.2
DQA1*05	22 (18.6)	39 (36.1)	0.41 (0.22–0.74)	0.0042	0.16	20.7	11 (14.1)	39 (36.1)	0.29 (0.14–0.61)	<0.001	0.030	8.4
DQB1*03	48 (39.3)	60 (55.0)	0.53 (0.31–0.89)	0.018	0.69	26.6	35 (43.2)	60 (55)	0.62 (0.35–1.11)	0.14	1	15.5
DQB1*06	105 (86.1)	81 (74.3)	2.14 (1.09–4.17)	0.03	1	37.0	75 (92.6)	81 (74.3)	4.32 (1.7–11.02)	0.0011	0.040	22.6
DRB1*03	25 (15.0)	41 (22.8)	0.6 (0.35–1.03)	0.075	1	25.0	14 (12.5)	41 (22.8)	0.48 (0.25–0.94)	0.032	1	11.4
DRB1*04	41 (24.1)	66 (36.7)	0.55 (0.35–0.87)	0.015	0.55	25.0	31 (27.4)	66 (36.7)	0.65 (0.39–1.09)	0.13	1	14.9
DRB1*08	28 (16.8)	10 (5.6)	3.42 (1.61–7.29)	<0.001	0.036	60.5	13 (11.6)	10 (5.6)	2.23 (0.94–5.28)	0.075	1	32.9
DRB1*15	140 (83.8)	100 (55.6)	4.15 (2.5–6.88)	<0.001	<0.001	43.3	102 (91.1)	100 (55.6)	8.16 (4–16.65)	<0.001	<0.001	27.7

	NAb development						Biologically relevant titers					
IFNβ-1b	No. NAb positive (%)	No. NAb negative (%)	OR (95% C.I.)	P^d^	P_C_ e	AR (%)^c^	No. BRT positive (%)	No. NAb negative (%)	OR (95% C.I.)	P^d^	P_C_ e	AR (%)^c^
**Treatment only** b	n/a	n/a	n/a	n/a	n/a	49.3	n/a	n/a	n/a	n/a	n/a	15.9
DRB1*04	66 (49.3)	16 (27.6)	2.55 (1.31–4.97)	0.0067	0.13	63.5	35 (57.4)	16 (27.6)	3.53 (1.64–7.61)	0.0015	0.029	28.2
DRB1*15	68 (51.9)	40 (69)	0.49 (0.25–0.93)	0.038	0.72	42.3	25 (42.4)	40 (69)	0.33 (0.16–0.71)	0.0052	0.098	10.4

a All HLA allele groups can be found in **Table S5**.

b Treatment only is the frequency of NAb development and biologically relevant titers for each treatment regardless of genotype.

c The absolute risk for each HLA allele group is calculated with Bayes' theorem and assessed based on frequency and impact on NAb development.

d Nominal *P*-values from Fishers exact test.

e Bonferroni corrected *P*-values (allele groups tested: 12 tests for i.m. IFNβ-1a, 19 tests for IFNβ-1b, 38 for s.c. IFNβ-1a).

Abbreviations: AR = absolute risk, C.I. = confidence interval, OR = odds ratio, IFNβ = interferon beta, n/a = not applicable, NAb = neutralizing antibodies.

For patients receiving s.c. IFNβ-1a an association was found for DRB1*15 with NAb positivity (OR 4.15; *P_c_*<0.001; AR 43.3%) and with biologically relevant titers (OR 8.16; *P_c_*<0.001; AR 27.7%) (Table 3). Associations were also found for carriage of DRB1*15 haplotype alleles C*07, B*07, DQB1*06 and DQA1*01 (Table 3), which consequently disappeared upon DRB1*15 stratification. In addition, DRB1*08 was found to be associated with NAb development (OR 3.42; *P_c_* = 0.036; AR 60.5%), however an association was not found within the smaller group of patients who developed biologically relevant titers (Table 3). Carriage of DQA1*05, which is commonly found on the same haplotype as DRB1*03, was associated with a decreased risk of developing biologically relevant titers (OR 0.29; *P_c_* = 0.032; AR 8.4%) (Table 3). Carriage of DRB1*04 and DQB1*03 were only nominally associated with NAb development and could represent the same signal because of extensive LD between these allele groups.

For patients receiving IFNβ-1b, DRB1*04 carriage was associated with development of biologically relevant titers (OR 3.53; *P* = 0.029; AR 28.2%) (Table 3). An association to HLA-DRB1*04 with all NAb positive patients regardless of titer level could not be replicated in the Swedish cohort. Since an association has previously been seen between DRB1*04 subtypes and NAb development, we extended our analysis for biologically relevant titers to also include DRB1*04 subtypes (Table S6). The OR for NAb development in DRB1*04:01 carriers compared to non-carriers was 3.43 (95% CI 1.48–7.93; *P* = 0.0039), while no association was found for DRB1*04:04 carriage (Table S6).

**Figure 1:**
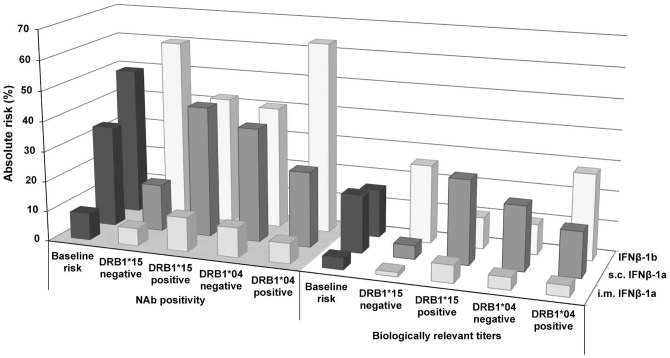
Absolute risk for development of NAbs and biologically relevant titers. Calculation of the absolute risk was used to estimate how DRB1*04 and DRB1*15 carriage impacts the risk for the outcomes of NAb positivity (bars on grey floor) and biologically relevant titers (bars on white floor) when adjusted for the baseline risk. Baseline risk was the frequency of development of NAb (dark grey bars, grey floor) and biologically relevant titers (dark grey bars, white floor) for each IFNβ preparation in the Swedish NAb registry. Compared to the baseline risks, DRB1*15 carriage increased the absolute risk for both outcomes in s.c. IFNβ-1a treated patients (grey bars) and to a lesser extent in patients receiving i.m. IFNβ-1a (light grey bars), whereas DRB1*15 carriage lowered the risk for both outcomes in patients receiving IFNβ-1b (white bars). In DRB1*04 carriers the reversed relationship was observed, with an increased absolute risk for both outcomes in IFNβ-1b treated patients. Moreover, the absolute risk for NAb positivity and biologically relevant titers was constantly lower for i.m. IFNβ-1a than for the two s.c. IFNβ preparations, regardless of whether patients were positive or negative for DRB1*04 and DRB1*15.

Although the influence of allele group carriage was taken into account when estimating the risk of developing NAbs for each specific treatment the treatment adjusted AR for NAb development was constantly higher for the two s.c. IFNβ preparations than for the i.m. IFNβ preparation, regardless of whether the respective risk alleles DRB1*15 for IFNβ-1a and DRB1*04 for IFNβ-1b were present or absent (Figure 1). For DRB1*15 carriers the AR of developing NAbs was higher if receiving s.c. IFNβ-1a (43% vs. 16%) and lower if receiving IFNβ-1b (60% vs. 42%) compared to DRB1*15 non-carriers, while the opposite was observed for DRB1*04 carrier-ship (Figure 1, Table 3). Similar results were observed for biologically relevant titers. Although the risk of NAbs for i.m. IFNβ-1a was much lower it followed the pattern of s.c. IFNβ-1a (Figure 1, Table 3).

### Binding prediction of HLA alleles to IFNβ peptides

The preparation-specific association to different HLA alleles was further investigated by analyzing differences in predicted binding of HLA alleles to IFNβ-1a and IFNβ-1b peptides. Especially the sites in IFNβ that differ between the two IFNβ molecules were investigated. The DRB1*04:01 allele, which was associated with an increased risk for NAbs in IFNβ-1b treated patients, was found to bind weakly to IFNβ-1b peptides containing Ser_17_ but not to IFNβ-1a peptides containing Cys_17_. Similar distinctions between the two IFNβ molecules could not be made for the alleles DRB1*04:04 and DRB1*15:01. Furthermore, DRB1*04:01 was predicted to bind with high affinity to peptides that included Asn_80_. However, this site is glycosylated in IFNβ-1a which could interfere with the binding to HLA alleles. Two IFNβ-1a peptides containing Met_1_ were found to bind strongly to HLA-C*07:01.

## Discussion

Preparation-related factors like dosage, injection site and frequency, aggregate content and genetic factors [9], [16], [20]–[22] may all contribute to IFNβ immunogenicity. In this cohort we have investigated the role of HLA gene carriage for the risk of developing overall NAbs and titers of NAbs high enough to affect the biological response to IFNβ. After stratification for the type of IFNβ molecules (IFNβ-1a and IFNβ-1b), we observed genetically distinct associations, DRB1*15 for IFNβ-1a and DRB1*04 for IFNβ-1b.

The risk for high NAb titers overall was 1.6-fold in patients carrying the MS risk haplotype DRB1*15, and for patients receiving IFNβ-1a the risk was increased up to 8-fold. The association between DRB1*15 and development of NAbs as well as high NAb titers is consistent with a previous study describing an increased proliferative response of CD4+ T cells against IFNβ epitopes in DRB1*15 carriers compared to non-carriers [18]. CD4+ T cell activation is important for induction of immunological memory and persistent B-cell responses [36] and the interaction between T cells and B cells through HLA class II suggests that HLA might influence this type of response.

There have been a few studies describing HLA associations with development of anti-drug antibodies against IFNβ [20]–[22]. In the study by Hoffmann *et al*., which included an initial study cohort and a validation cohort, DRB1*04:01 and DRB1*04:08 were found to be associated with an increased risk of developing antibodies against IFNβ in both groups [21]. These associations were further confirmed by Buck et al., using in an independent validation cohort [20]. Unlike these studies we found an association between NAb development and DRB1*15, whereas an association to DRB1*04 only was observed in patients treated with IFNβ-1b. Factors that could contribute to the discrepancy in genetic association between the studies are the differences in allele frequencies between the Swedish and the German populations regarding DRB1*15 (15.6% in Sweden vs. 14.2% in Germany) and DRB1*04 (18.9% vs 13.2% in Germany, according to http://www.allelefrequencies.net/default.asp; accessed December 13 in 2013) as well as the different proportions of patients receiving IFNβ-1a (78% vs 60%) and IFNβ-1b (22% vs 40%) in our cohort compared to the German cohort [21]. The fact that our cohort consisted of a higher proportion of IFNβ-1a users in which NAb development was associated to DRB1*15, and a lower proportion of IFNβ-1b users in which NAb development was associated to DRB1*04, could in part explain why we only observed an association to DRB1*15 in the total cohort of this study. Additionally, in the previous studies, both binding and neutralizing anti-drug antibodies against IFNβ were assessed together [20], [21] whereas in the present study only patients who were positive for neutralizing anti-drug antibodies against IFNβ were included. However, in contrast to the studies by Hoffmann *et al*. and Buck *et al*., we did not utilize a separate cohort for validation of our findings, which is a limitation of this study and thus these results need to be replicated in an independent cohort. The analysis of a possible association between DRB1*04:08 and NAb development could not be performed due to the origin of the HLA data. The problematic assignment of alleles to patients with imputed genotypes could potentially influence our results. However, 92% (33 of 36 patients) of the known DRB1*04:08 positive individuals from another cohort [30] were imputed as DRB1*04:01 when investigating this problem, and carriage of both DRB1*04:01 and DRB1*04:08 alleles has been shown to confer a higher risk of developing antibodies against IFNβ [20], [21].

This is the first report of distinct *DRB1* allele associations for the two different types of recombinant IFNβ molecules, DRB1*15 for IFNβ-1a and DRB1*04 for IFNβ-1b, indicating an IFNβ molecule dependent genetically determined risk to develop NAbs against IFNβ. There are several possible explanations that could account for differences in immunogenic potential. Sequence alterations, different formulation contents with a potential to evoke the immunological response, and different propensities to form aggregates due to the glycosylation status are all factors that might have a higher impact on NAb development. The different HLA associations found here might be explained by different binding affinities to the two types of IFNβ molecules to different HLA alleles. For instance, the increased risk for NAbs against IFNβ-1b in DRB1*04:01 carriers compared to non-carriers might to some extent be explained by the differential binding potential of this allele to IFNβ-1b compared to IFNβ-1a.

Binding prediction is based on the primary IFNβ sequence, but it is also possible that post-translational modifications such as glycosylation might interfere with peptide binding and presentation *in vivo*. Naturally processed peptides containing Asn-linked glycosylations can be presented on HLA class II molecules and these glycans can also alter the processing and presentation of peptides [37], [38]. We only observed an association with NAb to DRB1*04:01 in patients treated with IFNβ-1b, the non-glycosylated preparation. One could thus hypothesize that DRB1*04:01 carriage might lead to more efficient presentation of non-glycosylated peptides. This might be one explanation for the different HLA association to different IFNβ molecules.

Although HLA alleles influence the risk of NAbs within the treatment groups it is clear that NAb development in MS patients treated with IFNβ-1a is also influenced by several additional factors. Thus, IFNβ-1a injected intramuscularly is still the least immunogenic way of receiving IFNβ even for DRB1*15 carriers and none of the analyzed HLA allele groups lowered the risk of subcutaneously injected IFNβ to the level of intramuscularly injected IFNβ-1a. The skin harbors a high frequency of dendritic cells efficient in presenting antigens [39], easily provoked by repeated injections of IFNβ and potentially explaining higher immunogenicity of the subcutaneously administrated IFNβ products compared to the intramuscularly administered product.

Approximately 20% of IFNβ-treated patients develop NAb titers high enough to block treatment bio-efficacy. Since up to 60% of MS patients carry DRB1*15 and some 30% carry DRB1*04, and since the risk of high titer NAbs is increased several times (ORs 3–4) in carriers of respective risk alleles, it may well be relevant to base treatment decisions on HLA genotype. In fact, our empirical data suggest that DRB1*15 carriers would have a 35% risk of losing efficacy of s.c. IFNβ-1a but less than 10% on IFNβ-1b (provided they did not carry DRB1*04 on their other chromosome). The reverse risks would be seen for a DRB1*15-negative carrier, who carries DRB1*04, on IFNβ-1b. Although this study is based on over 900 patients the observed ORs show, however, large confidence intervals, making such clinical procedures somewhat premature. Extended studies on even larger patient cohorts are therefore warranted. Our findings may, though, have some bearing on other biological treatments for other diseases where blocking or neutralizing antibodies pose a problem.

## Conclusion

HLA class II genotype influences the risk of MS patients to develop NAbs against IFNβ-1a and IFNβ-1b. Further studies of the genetic risks for induction of anti-drug antibodies can provide important knowledge on possible immunological mechanisms triggered by regularly injected biopharmaceuticals.

## Supporting Information

Table S1 **Overview of percentage of patients with classical HLA data and patients with imputed HLA data for each gene.**
(DOC)

Table S2 **Allele frequency for all HLA genes analyzed for association to NAb development.**
(DOC)

Table S3 **Allele frequency for all HLA genes analyzed for association to development of biologically relevant titers.**
(DOC)

Table S4 **Frequency of NAb development and development of biologically relevant titers against each IFNβ preparation.**
(DOC)

Table S5 **Carrier frequency and absolute risk for HLA allele groups analyzed for association to NAb development and biologically relevant titers within each treatment group.**
(DOC)

Table S6 Association of DRB1*04 alleles in all patients and in the IFNβ-1b treated group of patients.
(DOC)
