# Supplementary material for: Human Leukocyte Antigen Genes and Interferon Beta Preparations Influence Risk of Developing Neutralizing Anti-Drug Antibodies in Multiple Sclerosis
Source: PLoS One. 2014 Mar 7;9(3):e90479. doi: 10.1371/journal.pone.0090479 (PMC3946519; doi:10.1371/journal.pone.0090479)
Supplement: Table S1 — Overview of percentage of patients with classical HLA data and patients with imputed HLA data for each gene. (DOC) [file pone.0090479.s001.doc]

**Table S1. Overview of percentage of patients with classical HLA data and patients with imputed HLA data for each gene.**

|  | **HLA-A, No. (%)** | **HLA-B, No. (%)** | **HLA-C, No. (%)** | **HLA-DRB1, No. (%)** | **HLA-DQA1, No. (%)** | **HLA-DQB1, No. (%)** | **High resolution DRB1*04, No. (%)^a^** |
| --- | --- | --- | --- | --- | --- | --- | --- |
| **Genotyped (frequency out of 903 patients)** | 884 (97.9) | 815 (90.3) | 810 (89.7) | 895 (99.1) | 598 (66.2) | 608 (67.3) | 252 (88.4) |
| **Have imputed data (frequency out of number of genotyped patients)** | 231 (26.1) | 500 (61.3) | 505 (62.3) | 218 (24.4) | 598 (100) | 608 (100) | 184 (73.0) |
| **Have classical data (frequency out of number of genotyped patients)** | 653 (73.9) | 315 (38.7) | 305 (37.7) | 677 (75.6) | 0 (0) | 0 (0) | 68 (27.0) |

^a^ Total DRB1*04 positive patients (N = 285).
