# Supplementary material for: Human Leukocyte Antigen Genes and Interferon Beta Preparations Influence Risk of Developing Neutralizing Anti-Drug Antibodies in Multiple Sclerosis
Source: PLoS One. 2014 Mar 7;9(3):e90479. doi: 10.1371/journal.pone.0090479 (PMC3946519; doi:10.1371/journal.pone.0090479)
Supplement: Table S2 — Allele frequency for all HLA genes analyzed for association to NAb development. (DOC) [file pone.0090479.s002.doc]

**Table S2. Allele frequency for all HLA genes analyzed for association to NAb development.**

| **HLA allele** | **No. NAb positive (%)** | **No. NAb negative (%)** | **Total cohort (%)** | **OR (95% C.I.)** | **P ^a^** | **P_C_ ^b^** |
| --- | --- | --- | --- | --- | --- | --- |
| **A*01** | 107 (15.0) | 168 (16.3) | 15.8 | 0.91 (0.7-1.18) | 0.50 | 1 |
| **A*02** | 206 (28.9) | 248 (24.0) | 26.0 | 1.28 (1.03-1.59) | 0.026 | 1 |
| **A*03** | 162 (22.7) | 234 (22.7) | 22.7 | 1 (0.8-1.26) | 1 | 1 |
| **A*11** | 34 (4.7) | 57 (5.5) | 5.2 | 0.85 (0.55-1.32) | 0.51 | 1 |
| **A*23** | 6 (0.8) | 8 (0.8) | 0.8 | 1.09 (0.38-3.14) | 1 | 1 |
| **A*24** | 57 (8.0) | 93 (9.0) | 8.6 | 0.88 (0.62-1.24) | 0.49 | 1 |
| **A*25** | 29 (4.1) | 35 (3.4) | 3.7 | 1.21 (0.73-1.99) | 0.52 | 1 |
| **A*26** | 16 (2.2) | 29 (2.8) | 2.6 | 0.79 (0.43-1.47) | 0.54 | 1 |
| **A*29** | 13 (1.8) | 20 (1.9) | 1.9 | 0.94 (0.46-1.9) | 1 | 1 |
| **A*30** | 4 (0.6) | 6 (0.6) | 0.6 | 0.96 (0.27-3.43) | 1 | 1 |
| **A*31** | 38 (5.3) | 41 (4.0) | 4.5 | 1.36 (0.87-2.14) | 0.20 | 1 |
| **A*32** | 20 (2.8) | 33 (3.2) | 3.0 | 0.87 (0.5-1.53) | 0.67 | 1 |
| **A*33** | 0 (0) | 4 (0.4) | 0.2 | N/A | 0.15 | 1 |
| **A*66** | 0 (0) | 1 (0.1) | 0.1 | N/A | 1 | 1 |
| **A*68** | 23 (3.2) | 55 (5.3) | 4.5 | 0.59 (0.36-0.97) | 0.045 | 1 |
| **B*07** | 185 (28.5) | 203 (22.2) | 24.8 | 1.4 (1.11-1.76) | 0.0052 | 0.40 |
| **B*08** | 65 (10.1) | 107 (11.8) | 11.1 | 0.84 (0.61-1.16) | 0.32 | 1 |
| **B*13** | 3 (0.5) | 2 (0.2) | 0.3 | 2.12 (0.35-12.69) | 0.65 | 1 |
| **B*14** | 12 (1.9) | 27 (3.0) | 2.5 | 0.62 (0.31-1.23) | 0.19 | 1 |
| **B*15** | 76 (11.7) | 120 (13.3) | 12.6 | 0.87 (0.64-1.18) | 0.39 | 1 |
| **B*18** | 34 (5.3) | 50 (5.5) | 5.4 | 0.95 (0.61-1.49) | 0.91 | 1 |
| **B*27** | 41 (6.4) | 57 (6.3) | 6.3 | 1.02 (0.67-1.54) | 0.92 | 1 |
| **B*35** | 23 (3.6) | 60 (6.7) | 5.4 | 0.52 (0.32-0.85) | 0.0084 | 0.64 |
| **B*37** | 12 (1.9) | 26 (2.9) | 2.5 | 0.64 (0.32-1.28) | 0.24 | 1 |
| **B*38** | 1 (0.2) | 5 (0.6) | 0.4 | 0.28 (0.03-2.4) | 0.41 | 1 |
| **B*39** | 13 (2.0) | 7 (0.8) | 1.3 | 2.65 (1.05-6.67) | 0.040 | 1 |

**Table S2. Allele frequency for all HLA genes analyzed for association to NAb development (continued).**

| **HLA allele** | **No. NAb positive (%)** | **No. NAb negative (%)** | **Total cohort (%)** | **OR (95% C.I.)** | **P ^a^** | **P_C_ ^b^** |
| --- | --- | --- | --- | --- | --- | --- |
| **B*40** | 53 (8.2) | 63 (7.0) | 7.5 | 1.19 (0.82-1.75) | 0.38 | 1 |
| **B*41** | 3 (0.5) | 7 (0.8) | 0.6 | 0.6 (0.16-2.33) | 0.54 | 1 |
| **B*44** | 58 (9.1) | 77 (8.6) | 8.8 | 1.07 (0.75-1.52) | 0.78 | 1 |
| **B*45** | 3 (0.5) | 5 (0.6) | 0.5 | 0.84 (0.2-3.54) | 1 | 1 |
| **B*47** | 4 (0.6) | 3 (0.3) | 0.5 | 1.88 (0.42-8.43) | 0.46 | 1 |
| **B*49** | 0 (0) | 6 (0.7) | 0.4 | N/A | 0.045 | 1 |
| **B*50** | 3 (0.5) | 3 (0.3) | 0.4 | 1.41 (0.28-7) | 0.70 | 1 |
| **B*51** | 34 (5.3) | 48 (5.3) | 5.3 | 1 (0.64-1.57) | 1 | 1 |
| **B*52** | 2 (0.3) | 5 (0.6) | 0.5 | 0.56 (0.11-2.9) | 0.71 | 1 |
| **B*53** | 0 (0) | 2 (0.2) | 0.1 | N/A | 0.51 | 1 |
| **B*55** | 3 (0.5) | 8 (0.9) | 0.7 | 0.53 (0.14-1.99) | 0.38 | 1 |
| **B*56** | 7 (1.1) | 4 (0.4) | 0.7 | 2.48 (0.72-8.5) | 0.22 | 1 |
| **B*57** | 16 (2.5) | 21 (2.3) | 2.4 | 1.07 (0.56-2.07) | 0.87 | 1 |
| **B*58** | 3 (0.5) | 3 (0.3) | 0.4 | 1.41 (0.28-7) | 0.70 | 1 |
| **C*01** | 21 (3.2) | 28 (3.0) | 3.1 | 1.06 (0.6-1.89) | 0.88 | 1 |
| **C*02** | 39 (5.9) | 55 (5.9) | 5.9 | 1 (0.65-1.52) | 1 | 1 |
| **C*03** | 124 (18.9) | 182 (19.6) | 19.3 | 0.96 (0.74-1.24) | 0.75 | 1 |
| **C*04** | 36 (5.5) | 74 (8.0) | 7.0 | 0.67 (0.44-1.01) | 0.057 | 1 |
| **C*05** | 41 (6.3) | 58 (6.3) | 6.3 | 1 (0.66-1.51) | 1 | 1 |
| **C*06** | 41 (6.3) | 58 (6.3) | 6.3 | 1 (0.66-1.51) | 1 | 1 |
| **C*07** | 279 (42.4) | 352 (38.0) | 39.8 | 1.2 (0.98-1.47) | 0.086 | 1 |
| **C*08** | 12 (1.8) | 28 (3.0) | 2.5 | 0.6 (0.3-1.19) | 0.15 | 1 |
| **C*12** | 22 (3.4) | 39 (4.2) | 3.9 | 0.79 (0.47-1.35) | 0.43 | 1 |
| **C*14** | 6 (0.9) | 8 (0.9) | 0.9 | 1.06 (0.37-3.08) | 1 | 1 |
| **C*15** | 20 (3.1) | 28 (3.0) | 3.0 | 1.01 (0.57-1.81) | 1 | 1 |
| **C*16** | 16 (2.4) | 11 (1.2) | 1.7 | 2.09 (0.96-4.53) | 0.075 | 1 |
| **C*17** | 3 (0.5) | 7 (0.8) | 0.6 | 0.61 (0.16-2.35) | 0.54 | 1 |

**Table S2. Allele frequency for all HLA genes analyzed for association to NAb development (continued).**

| **HLA allele** | **No. NAb positive (%)** | **No. NAb negative (%)** | **Total cohort (%)** | **OR (95% C.I.)** | **P ^a^** | **P_C_ ^b^** |
| --- | --- | --- | --- | --- | --- | --- |
| **DQA1*01** | 299 (58.2) | 387 (56.7) | 57.4 | 1.06 (0.84-1.34) | 0.64 | 1 |
| **DQA1*02** | 32 (6.2) | 36 (5.3) | 5.7 | 1.19 (0.73-1.95) | 0.53 | 1 |
| **DQA1*03** | 90 (17.5) | 112 (16.4) | 16.9 | 1.08 (0.8-1.47) | 0.64 | 1 |
| **DQA1*04** | 32 (6.2) | 25 (3.7) | 4.8 | 1.75 (1.02-2.98) | 0.054 | 1 |
| **DQA1*05** | 61 (11.9) | 122 (17.9) | 15.3 | 0.62 (0.44-0.86) | 0.0045 | 0.34 |
| **DQB1*02** | 61 (11.4) | 99 (14.6) | 13.2 | 0.75 (0.54-1.06) | 0.11 | 1 |
| **DQB1*03** | 144 (26.9) | 176 (25.9) | 26.3 | 1.05 (0.81-1.36) | 0.74 | 1 |
| **DQB1*04** | 31 (5.8) | 25 (3.7) | 4.6 | 1.61 (0.94-2.76) | 0.10 | 1 |
| **DQB1*05** | 31 (5.8) | 76 (11.2) | 8.8 | 0.49 (0.32-0.75) | 0.0010 | 0.079 |
| **DQB1*06** | 269 (50.2) | 304 (44.7) | 47.1 | 1.25 (0.99-1.56) | 0.064 | 1 |
| **DRB1*01** | 34 (4.8) | 85 (8.1) | 6.8 | 0.58 (0.38-0.87) | 0.0087 | 0.66 |
| **DRB1*03** | 56 (8.0) | 124 (11.9) | 10.3 | 0.65 (0.46-0.9) | 0.010 | 0.77 |
| **DRB1*04** | 124 (17.4) | 171 (16.3) | 16.8 | 1.08 (0.83-1.39) | 0.60 | 1 |
| **DRB1*07** | 43 (6.1) | 52 (5.0) | 5.4 | 1.25 (0.83-1.9) | 0.33 | 1 |
| **DRB1*08** | 43 (6.1) | 52 (5.0) | 5.4 | 1.25 (0.83-1.9) | 0.33 | 1 |
| **DRB1*09** | 1 (0.1) | 7 (0.7) | 0.5 | 0.21 (0.03-1.73) | 0.15 | 1 |
| **DRB1*10** | 1 (0.1) | 4 (0.4) | 0.3 | 0.37 (0.04-3.34) | 0.65 | 1 |
| **DRB1*11** | 23 (3.3) | 47 (4.5) | 4.0 | 0.72 (0.43-1.19) | 0.22 | 1 |
| **DRB1*12** | 5 (0.7) | 12 (1.1) | 1.0 | 0.62 (0.22-1.77) | 0.46 | 1 |
| **DRB1*13** | 78 (11.1) | 127 (12.1) | 11.7 | 0.91 (0.67-1.23) | 0.54 | 1 |
| **DRB1*14** | 8 (1.1) | 9 (0.9) | 1.0 | 1.33 (0.51-3.47) | 0.62 | 1 |
| **DRB1*15** | 291 (41.6) | 348 (33.3) | 36.6 | 1.43 (1.17-1.74) | < 0.001 | 0.036 |
| **DRB1*16** | 4 (0.6) | 9 (0.9) | 0.7 | 0.66 (0.2-2.16) | 0.58 | 1 |

^a^ Nominal *P*-values from Fishers exact test

^b^ Bonferroni corrected *P*-values (76 allele groups tested)

Abbreviations: C.I.=confidence interval, N/A=not available, NAb=neutralizing antibodies, OR=odds ratio
