# Supplementary material for: Human Leukocyte Antigen Genes and Interferon Beta Preparations Influence Risk of Developing Neutralizing Anti-Drug Antibodies in Multiple Sclerosis
Source: PLoS One. 2014 Mar 7;9(3):e90479. doi: 10.1371/journal.pone.0090479 (PMC3946519; doi:10.1371/journal.pone.0090479)
Supplement: Table S3 — Allele frequency for all HLA genes analyzed for association to development of biologically relevant titers. (DOC) [file pone.0090479.s003.doc]

**Table S3. Allele frequency for all HLA genes analyzed for association to development of biologically relevant titers.**

| **HLA allele** | **No. NAb positive (%)** | **No. NAb negative (%)** | **Total cohort (%)** | **OR (95% C.I.)** | **P ^a^** | **P_C_ ^b^** |
| --- | --- | --- | --- | --- | --- | --- |
| **A*01** | 69 (16.7) | 168 (16.3) | 16.4 | 1.04 (0.76-1.41) | 0.81 | 1 |
| **A*02** | 119 (28.9) | 248 (24.0) | 25.4 | 1.28 (0.99-1.66) | 0.061 | 1 |
| **A*03** | 93 (22.6) | 234 (22.7) | 22.6 | 0.99 (0.76-1.31) | 1 | 1 |
| **A*11** | 18 (4.3) | 57 (5.5) | 5.2 | 0.78 (0.45-1.34) | 0.43 | 1 |
| **A*23** | 4 (1.0) | 8 (0.8) | 0.8 | 1.26 (0.38-4.19) | 0.75 | 1 |
| **A*24** | 35 (8.5) | 93 (9.0) | 8.9 | 0.94 (0.62-1.41) | 0.84 | 1 |
| **A*25** | 18 (4.4) | 35 (3.4) | 3.7 | 1.3 (0.73-2.33) | 0.36 | 1 |
| **A*26** | 9 (2.2) | 29 (2.8) | 2.6 | 0.77 (0.36-1.65) | 0.59 | 1 |
| **A*29** | 8 (1.9) | 20 (1.9) | 1.9 | 1 (0.44-2.29) | 1 | 1 |
| **A*30** | 1 (0.2) | 6 (0.6) | 0.5 | 0.42 (0.05-3.47) | 0.68 | 1 |
| **A*31** | 22 (5.3) | 41 (4.0) | 4.4 | 1.36 (0.8-2.32) | 0.26 | 1 |
| **A*32** | 8 (1.9) | 33 (3.2) | 2.8 | 0.6 (0.28-1.31) | 0.22 | 1 |
| **A*33** | 0 (0) | 4 (0.4) | 0.3 | N/A | 0.58 | 1 |
| **A*66** | 0 (0) | 1 (0.1) | 0.1 | N/A | 1 | 1 |
| **A*68** | 9 (2.2) | 55 (5.3) | 4.4 | 0.4 (0.19-0.81) | 0.0070 | 0.53 |
| **B*07** | 118 (31.4) | 203 (22.2) | 24.8 | 1.61 (1.23-2.1) | < 0.001 | 0.050 |
| **B*08** | 39 (10.5) | 107 (11.8) | 11.4 | 0.87 (0.59-1.29) | 0.56 | 1 |
| **B*13** | 2 (0.5) | 2 (0.2) | 0.3 | 2.44 (0.34-17.39) | 0.58 | 1 |
| **B*14** | 8 (2.2) | 27 (3.0) | 2.8 | 0.72 (0.32-1.59) | 0.46 | 1 |
| **B*15** | 44 (11.8) | 120 (13.3) | 12.8 | 0.87 (0.6-1.26) | 0.52 | 1 |
| **B*18** | 19 (5.1) | 50 (5.5) | 5.4 | 0.92 (0.54-1.59) | 0.89 | 1 |
| **B*27** | 23 (6.2) | 57 (6.3) | 6.3 | 0.99 (0.6-1.63) | 1 | 1 |
| **B*35** | 11 (3.0) | 60 (6.7) | 5.6 | 0.43 (0.22-0.83) | 0.010 | 0.76 |
| **B*37** | 8 (2.2) | 26 (2.9) | 2.7 | 0.74 (0.33-1.66) | 0.57 | 1 |
| **B*38** | 1 (0.3) | 5 (0.6) | 0.5 | 0.49 (0.06-4.17) | 0.68 | 1 |
| **B*39** | 7 (1.9) | 7 (0.8) | 1.1 | 2.46 (0.86-7.06) | 0.13 | 1 |

**Table S3. Allele frequency for all HLA genes analyzed for association to development of biologically relevant titers (continued).**

| **HLA allele** | **No. NAb positive (%)** | **No. NAb negative (%)** | **Total cohort (%)** | **OR (95% C.I.)** | **P ^a^** | **P_C_ ^b^** |
| --- | --- | --- | --- | --- | --- | --- |
| **B*40** | 26 (7.0) | 63 (7.0) | 7.0 | 1.01 (0.63-1.62) | 1 | 1 |
| **B*41** | 2 (0.5) | 7 (0.8) | 0.7 | 0.69 (0.14-3.35) | 1 | 1 |
| **B*44** | 34 (9.2) | 77 (8.6) | 8.7 | 1.08 (0.71-1.65) | 0.74 | 1 |
| **B*45** | 2 (0.5) | 5 (0.6) | 0.6 | 0.97 (0.19-5.04) | 1 | 1 |
| **B*47** | 2 (0.5) | 3 (0.3) | 0.4 | 1.63 (0.27-9.77) | 0.63 | 1 |
| **B*49** | 0 (0) | 6 (0.7) | 0.5 | N/A | 0.19 | 1 |
| **B*50** | 1 (0.3) | 3 (0.3) | 0.3 | 0.81 (0.08-7.82) | 1 | 1 |
| **B*51** | 11 (3.0) | 48 (5.3) | 4.6 | 0.55 (0.28-1.06) | 0.078 | 1 |
| **B*52** | 2 (0.5) | 5 (0.6) | 0.6 | 0.97 (0.19-5.04) | 1 | 1 |
| **B*53** | 0 (0) | 2 (0.2) | 0.2 | N/A | 1 | 1 |
| **B*55** | 1 (0.3) | 8 (0.9) | 0.7 | 0.3 (0.04-2.43) | 0.46 | 1 |
| **B*56** | 3 (0.8) | 4 (0.4) | 0.6 | 1.83 (0.41-8.22) | 0.42 | 1 |
| **B*57** | 10 (2.7) | 21 (2.3) | 2.4 | 1.16 (0.54-2.49) | 0.69 | 1 |
| **B*58** | 2 (0.5) | 3 (0.3) | 0.4 | 1.63 (0.27-9.77) | 0.63 | 1 |
| **C*01** | 11 (2.9) | 28 (3.0) | 3.0 | 0.97 (0.48-1.97) | 1 | 1 |
| **C*02** | 19 (5.0) | 55 (5.9) | 5.7 | 0.84 (0.49-1.43) | 0.68 | 1 |
| **C*03** | 67 (17.8) | 182 (19.6) | 19.1 | 0.89 (0.65-1.22) | 0.79 | 1 |
| **C*04** | 21 (5.6) | 74 (8.0) | 7.3 | 0.69 (0.42-1.13) | 0.11 | 1 |
| **C*05** | 21 (5.6) | 58 (6.3) | 6.1 | 0.89 (0.53-1.49) | 0.89 | 1 |
| **C*06** | 25 (6.6) | 58 (6.3) | 6.4 | 1.07 (0.66-1.73) | 0.79 | 1 |
| **C*07** | 175 (46.5) | 352 (38) | 40.5 | 1.42 (1.11-1.81) | 0.015 | 1 |
| **C*08** | 8 (2.1) | 28 (3.0) | 2.8 | 0.7 (0.32-1.55) | 0.45 | 1 |
| **C*12** | 14 (3.7) | 39 (4.2) | 4.1 | 0.88 (0.47-1.65) | 0.75 | 1 |
| **C*14** | 0 (0) | 8 (0.9) | 0.6 | N/A | 0.11 | 1 |
| **C*15** | 8 (2.1) | 28 (3.0) | 2.8 | 0.7 (0.32-1.55) | 0.43 | 1 |
| **C*16** | 8 (2.1) | 11 (1.2) | 1.5 | 1.82 (0.73-4.56) | 0.20 | 1 |
| **C*17** | 2 (0.5) | 7 (0.8) | 0.7 | 0.71 (0.15-3.41) | 1 | 1 |

**Table S3. Allele frequency for all HLA genes analyzed for association to development of biologically relevant titers (continued).**

| **HLA allele** | **No. NAb positive (%)** | **No. NAb negative (%)** | **Total cohort (%)** | **OR (95% C.I.)** | **P ^a^** | **P_C_ ^b^** |
| --- | --- | --- | --- | --- | --- | --- |
| **DQA1*01** | 175 (59.5) | 387 (56.7) | 57.6 | 1.12 (0.85-1.48) | 0.44 | 1 |
| **DQA1*02** | 21 (7.1) | 36 (5.3) | 5.8 | 1.38 (0.79-2.41) | 0.30 | 1 |
| **DQA1*03** | 57 (19.4) | 112 (16.4) | 17.3 | 1.22 (0.86-1.74) | 0.27 | 1 |
| **DQA1*04** | 13 (4.4) | 25 (3.7) | 3.9 | 1.22 (0.61-2.41) | 0.59 | 1 |
| **DQA1*05** | 28 (9.5) | 122 (17.9) | 15.4 | 0.48 (0.31-0.75) | < 0.001 | 0.052 |
| **DQB1*02** | 36 (11.6) | 99 (14.6) | 13.6 | 0.77 (0.51-1.16) | 0.23 | 1 |
| **DQB1*03** | 84 (27.1) | 176 (25.9) | 26.3 | 1.06 (0.79-1.44) | 0.70 | 1 |
| **DQB1*04** | 13 (4.2) | 25 (3.7) | 3.8 | 1.15 (0.58-2.27) | 0.72 | 1 |
| **DQB1*05** | 17 (5.5) | 76 (11.2) | 9.4 | 0.46 (0.27-0.79) | 0.0046 | 0.35 |
| **DQB1*06** | 160 (51.6) | 304 (44.7) | 46.9 | 1.32 (1.01-1.73) | 0.047 | 1 |
| **DRB1*01** | 20 (4.9) | 85 (8.1) | 7.2 | 0.59 (0.36-0.97) | 0.041 | 1 |
| **DRB1*03** | 32 (7.9) | 124 (11.9) | 10.8 | 0.64 (0.43-0.96) | 0.030 | 1 |
| **DRB1*04** | 79 (19.2) | 171 (16.3) | 17.1 | 1.21 (0.9-1.63) | 0.22 | 1 |
| **DRB1*07** | 28 (6.9) | 52 (5.0) | 5.5 | 1.42 (0.89-2.29) | 0.16 | 1 |
| **DRB1*08** | 19 (4.7) | 52 (5.0) | 4.9 | 0.94 (0.55-1.62) | 0.89 | 1 |
| **DRB1*09** | 0 (0) | 7 (0.7) | 0.5 | N/A | 0.20 | 1 |
| **DRB1*10** | 0 (0) | 4 (0.4) | 0.3 | N/A | 0.58 | 1 |
| **DRB1*11** | 5 (1.2) | 47 (4.5) | 3.6 | 0.27 (0.11-0.68) | 0.0023 | 0.18 |
| **DRB1*12** | 1 (0.2) | 12 (1.1) | 0.9 | 0.21 (0.03-1.65) | 0.13 | 1 |
| **DRB1*13** | 40 (9.9) | 127 (12.1) | 11.5 | 0.8 (0.55-1.16) | 0.27 | 1 |
| **DRB1*14** | 5 (1.2) | 9 (0.9) | 1.0 | 1.44 (0.48-4.34) | 0.55 | 1 |
| **DRB1*15** | 178 (44.1) | 348 (33.3) | 36.3 | 1.58 (1.25-2) | < 0.001 | 0.012 |
| **DRB1*16** | 2 (0.5) | 9 (0.9) | 0.8 | 0.57 (0.12-2.67) | 0.74 | 1 |

^a^ Nominal *P*-values from Fishers exact test

^b^ Bonferroni corrected *P*-values (76 allele groups tested)

Abbreviations: C.I.=confidence interval, N/A=not available, NAb=neutralizing antibodies, OR=odds ratio
