# Supplementary material for: Human Leukocyte Antigen Genes and Interferon Beta Preparations Influence Risk of Developing Neutralizing Anti-Drug Antibodies in Multiple Sclerosis
Source: PLoS One. 2014 Mar 7;9(3):e90479. doi: 10.1371/journal.pone.0090479 (PMC3946519; doi:10.1371/journal.pone.0090479)
Supplement: Table S4 — Frequency of NAb development and development of biologically relevant titers against each IFNβ preparation. (DOC) [file pone.0090479.s004.doc]

**Table S4. Frequency of NAb development and development of biologically relevant titers against each IFNβ** preparation.

| **Preparation** | **No. total treated** | **No. NAb positive (%) ^a^** | **No. biologically relevant titers (%) ^a^** |
| --- | --- | --- | --- |
| **IFNβ-1a i.m.** | 949 | 85 (9.0) | 37 (3.9) |
| **IFNβ-1a s.c.** | 922 | 310 (33.6) | 175 (19.0) |
| **IFNβ-1b** | 598 | 295 (49.3) | 95 (15.9) |

^a^ The calculated frequency of positivity for NAbs or biologically relevant titers for each preparation based on the numbers for the whole NAb registry regardless of genotype information (positive patients having at least one positive sample otherwise considered negative).

Abbreviations: IFNβ=interferon beta, i.m.=intramuscular, s.c.=subcutaneous, NAb=neutralizing antibodies
