# Supplementary material for: Human Leukocyte Antigen Genes and Interferon Beta Preparations Influence Risk of Developing Neutralizing Anti-Drug Antibodies in Multiple Sclerosis
Source: PLoS One. 2014 Mar 7;9(3):e90479. doi: 10.1371/journal.pone.0090479 (PMC3946519; doi:10.1371/journal.pone.0090479)
Supplement: Table S5 — Carrier frequency and absolute risk for HLA allele groups analyzed for association to NAb development and biologically relevant titers within each treatment group. (DOC) [file pone.0090479.s005.doc]

Table S5. Carrier frequency and absolute risk for HLA allele groups analyzed for association to NAb development and biologically relevant titers within each treatment group.

|  | **NAb development** | | | | | | **Biologically relevant titers** | | | | | |
| --- | --- | --- | --- | --- | --- | --- | --- | --- | --- | --- | --- | --- |
| **i.m. IFNβ-1a** | **No. NAb positive (%)** | **No. NAb negative (%)** | **OR (95% C.I.)** | **P ^c^** | **P_C_ ^d^** | **AR % ^b^** | **No. BRT positive (%)** | **No. NAb negative (%)** | **OR (95% C.I.)** | **P ^c^** | **P_C_ ^d^** | **AR % ^b^** |
| **Treatment only ^a^** | n/a | n/a | n/a | n/a | n/a | 9.0 | n/a | n/a | n/a | n/a | n/a | 3.9 |
| **A*01** | 16 (34.0) | 81 (28.5) | 1.29 (0.67-2.49) | 0.49 | 1 | 10.5 | 11 (42.3) | 81 (28.5) | 1.84 (0.81-4.17) | 0.18 | 1 | 5.7 |
| **A*02** | 18 (38.3) | 106 (37.3) | 1.04 (0.55-1.97) | 1 | 1 | 9.2 | 10 (38.5) | 106 (37.3) | 1.05 (0.46-2.4) | 1 | 1 | 4.0 |
| **A*03** | 22 (46.8) | 125 (44.0) | 1.12 (0.6-2.08) | 0.75 | 1 | 9.5 | 10 (38.5) | 125 (44.0) | 0.8 (0.35-1.81) | 0.68 | 1 | 3.4 |
| **B*07** | 20 (47.6) | 105 (41.0) | 1.31 (0.68-2.52) | 0.50 | 1 | 10.3 | 11 (45.8) | 105 (41.0) | 1.2 (0.53-2.82) | 0.67 | 1 | 4.3 |
| **B*08** | 12 (30.0) | 55 (21.7) | 1.55 (0.74-3.25) | 0.31 | 1 | 12.0 | N/A | N/A | N/A | N/A | N/A | N/A |
| **C*03** | 17 (37.8) | 92 (35.7) | 1.1 (0.57-2.11) | 0.87 | 1 | 9.4 | 10 (40.0) | 92 (35.7) | 1.2 (0.52-2.79) | 0.67 | 1 | 4.4 |
| **C*07** | 30 (68.2) | 156 (60.7) | 1.39 (0.7-2.74) | 0.40 | 1 | 10.0 | 17 (70.8) | 156 (60.7) | 1.57 (0.63-3.93) | 0.39 | 1 | 4.5 |
| **DQA1*01** | 32 (97.0) | 172 (86.4) | 5.02 (0.66-38.3) | 0.14 | 1 | 9.9 | 19 (100.0) | 172 (86.4) | N/A | 0.14 | 1 | 4.5 |
| **DQB1*06** | 31 (93.9) | 141 (71.9) | 6.05 (1.4-26.13) | 0.0046 | 0.055 | 11.4 | 17 (94.4) | 141 (71.9) | 6.63 (0.86-51.04) | 0.047 | 0.57 | 5.1 |
| **DRB1*04** | 10 (21.3) | 83 (29.1) | 0.66 (0.31-1.38) | 0.30 | 1 | 6.7 | N/A | N/A | N/A | N/A | N/A | N/A |
| **DRB1*13** | 17 (36.2) | 68 (23.7) | 1.81 (0.94-3.48) | 0.10 | 1 | 13.0 | 11 (42.3) | 68 (23.7) | 2.34 (1.03-5.34) | 0.057 | 0.68 | 6.7 |
| **DRB1*15** | 34 (72.3) | 159 (55.8) | 2.07 (1.05-4.09) | 0.038 | 0.46 | 11.3 | 22 (84.6) | 159 (55.8) | 4.36 (1.46-12.97) | 0.0036 | 0.044 | 5.8 |
|  | **NAb development** | | | | | | **Biologically relevant titers** | | | | | |
| **s.c. IFNβ-1a** | **No. NAb positive (%)** | **No. NAb negative (%)** | **OR (95% C.I.)** | **P ^c^** | **P_C_ ^d^** | **AR % ^b^** | **No. BRT positive (%)** | **No. NAb negative (%)** | **OR (95% C.I.)** | **P ^c^** | **P_C_ ^d^** | **AR % ^b^** |
| **Treatment only ^a^** | n/a | n/a | n/a | n/a | n/a | 33.6 | n/a | n/a | n/a | n/a | n/a | 19.0 |
| **A*01** | 46 (26.9) | 60 (34.3) | 0.71 (0.45-1.12) | 0.16 | 1 | 28.4 | 28 (24.8) | 60 (34.3) | 0.63 (0.37-1.07) | 0.091 | 1 | 14.5 |
| **A*02** | 86 (50.3) | 79 (45.1) | 1.23 (0.81-1.88) | 0.39 | 1 | 36.1 | 55 (48.7) | 79 (45.1) | 1.15 (0.72-1.85) | 0.63 | 1 | 20.2 |
| **A*03** | 68 (39.8) | 57 (32.6) | 1.37 (0.88-2.12) | 0.18 | 1 | 38.2 | 50 (44.2) | 57 (32.6) | 1.64 (1.01-2.68) | 0.047 | 1 | 24.1 |

Table S5. Carrier frequency and absolute risk for HLA allele groups analyzed for association to NAb development and biologically relevant titers within each treatment group (continued).

|  | **NAb development** | | | | | | **Biologically relevant titers** | | | | | |
| --- | --- | --- | --- | --- | --- | --- | --- | --- | --- | --- | --- | --- |
| **s.c. IFNβ-1a** | **No. NAb positive (%)** | **No. NAb negative (%)** | **OR (95% C.I.)** | **P ^c^** | **P_C_ ^d^** | **AR % ^b^** | **No. BRT positive (%)** | **No. NAb negative (%)** | **OR (95% C.I.)** | **P ^c^** | **P_C_ ^d^** | **AR % ^b^** |
| **A*11** | 16 (9.4) | 16 (9.1) | 1.03 (0.5-2.12) | 1 | 1 | 34.1 | N/A | N/A | N/A | N/A | N/A | N/A |
| **A*24** | 26 (15.2) | 26 (14.9) | 1.03 (0.57-1.85) | 1 | 1 | 40.9 | 20 (17.7) | 26 (14.9) | 1.23 (0.65-2.33) | 0.52 | 1 | 28.2 |
| **A*25** | 16 (9.4) | 12 (6.9) | 1.4 (0.64-3.06) | 0.43 | 1 | 34.1 | 13 (11.5) | 12 (6.9) | 1.77 (0.78-4.02) | 0.20 | 1 | 21.8 |
| **A*31** | 20 (11.7) | 13 (7.4) | 1.65 (0.79-3.43) | 0.20 | 1 | 44.4 | 15 (13.3) | 13 (7.4) | 1.91 (0.87-4.18) | 0.11 | 1 | 29.5 |
| **A*68** | 16 (9.4) | 19 (10.9) | 0.85 (0.42-1.71) | 0.72 | 1 | 30.4 | N/A | N/A | N/A | N/A | N/A | N/A |
| **B*07** | 86 (54.8) | 56 (37.1) | 2.06 (1.3-3.24) | 0.0021 | 0.08 | 42.8 | 66 (64.7) | 56 (37.1) | 3.11 (1.84-5.25) | <0.001 | <0.001 | 29.0 |
| **B*08** | 26 (16.7) | 30 (20.4) | 0.78 (0.44-1.4) | 0.46 | 1 | 29.3 | 16 (15.7) | 30 (20.4) | 0.73 (0.37-1.42) | 0.41 | 1 | 15.3 |
| **B*15** | 29 (18.6) | 35 (23.8) | 0.73 (0.42-1.27) | 0.32 | 1 | 28.3 | 18 (17.6) | 35 (23.8) | 0.69 (0.36-1.29) | 0.27 | 1 | 14.8 |
| **B*18** | 16 (10.3) | 18 (12.2) | 0.83 (0.4-1.69) | 0.72 | 1 | 29.9 | 12 (11.9) | 18 (12.2) | 0.97 (0.44-2.11) | 1 | 1 | 18.5 |
| **B*27** | 17 (11.0) | 14 (9.5) | 1.18 (0.56-2.49) | 0.71 | 1 | 37.0 | 11 (10.9) | 14 (9.5) | 1.17 (0.51-2.69) | 0.83 | 1 | 21.2 |
| **B*40** | 26 (16.7) | 22 (14.9) | 1.15 (0.62-2.13) | 0.75 | 1 | 36.2 | 12 (11.9) | 22 (14.9) | 0.77 (0.36-1.64) | 0.58 | 1 | 15.8 |
| **B*44** | 24 (15.5) | 32 (21.8) | 0.66 (0.37-1.18) | 0.18 | 1 | 26.5 | 16 (15.8) | 32 (21.8) | 0.68 (0.35-1.31) | 0.26 | 1 | 14.6 |
| **B*51** | 21 (13.5) | 14 (9.5) | 1.5 (0.73-3.07) | 0.29 | 1 | 42.0 | N/A | N/A | N/A | N/A | N/A | N/A |
| **C*02** | 15 (9.6) | 16 (10.5) | 0.9 (0.43-1.9) | 0.85 | 1 | 31.6 | N/A | N/A | N/A | N/A | N/A | N/A |
| **C*03** | 57 (36.5) | 51 (33.3) | 1.15 (0.72-1.84) | 0.63 | 1 | 35.7 | 32 (31.4) | 51 (33.3) | 0.91 (0.54-1.56) | 0.79 | 1 | 18.1 |
| **C*04** | 14 (9.0) | 21 (13.8) | 0.62 (0.3-1.26) | 0.21 | 1 | 24.8 | N/A | N/A | N/A | N/A | N/A | N/A |
| **C*05** | 14 (9.0) | 19 (12.5) | 0.69 (0.33-1.43) | 0.36 | 1 | 26.7 | N/A | N/A | N/A | N/A | N/A | N/A |
| **C*06** | 21 (13.4) | 20 (13.2) | 1.02 (0.53-1.97) | 1 | 1 | 34.0 | 16 (15.5) | 20 (13.2) | 1.21 (0.6-2.47) | 0.59 | 1 | 21.7 |
| **C*07** | 112 (70.9) | 91 (59.9) | 1.63 (1.02-2.62) | 0.043 | 1 | 37.5 | 81 (78.6) | 91 (59.9) | 2.47 (1.39-4.37) | 0.0017 | 0.066 | 23.5 |
| **C*12** | 11 (7.1) | 16 (10.5) | 0.65 (0.29-1.44) | 0.32 | 1 | 25.3 | 10 (9.8) | 16 (10.5) | 0.92 (0.4-2.13) | 1 | 1 | 17.9 |
| **C*15** | 14 (9.0) | 10 (6.6) | 1.4 (0.6-3.26) | 0.53 | 1 | 40.9 | N/A | N/A | N/A | N/A | N/A | N/A |
| **DQA1*01** | 107 (90.7) | 89 (82.4) | 2.08 (0.94-4.59) | 0.079 | 1 | 35.8 | 74 (94.9) | 89 (82.4) | 3.95 (1.29-12.12) | 0.012 | 0.47 | 21.2 |
| **DQA1*02** | 14 (11.9) | 14 (13.0) | 0.9 (0.41-2.0) | 0.84 | 1 | 31.7 | 10 (12.8) | 14 (13.0) | 0.99 (0.41-2.36) | 1 | 1 | 18.8 |
| **DQA1*03** | 32 (27.1) | 41 (38.0) | 0.61 (0.35-1.07) | 0.089 | 1 | 26.6 | 25 (32.1) | 41 (38.0) | 0.77 (0.42-1.43) | 0.44 | 1 | 16.5 |

**Table S5. Carrier frequency and absolute risk for HLA allele groups analyzed for association to NAb development and biologically relevant titers within each treatment group (continued).**

|  | **NAb development** | | | | | | | | **Biologically relevant titers** | | | | | |
| --- | --- | --- | --- | --- | --- | --- | --- | --- | --- | --- | --- | --- | --- | --- |
| **s.c. IFNβ-1a** | **No. NAb positive (%)** | | **No. NAb negative (%)** | | **OR (95% C.I.)** | **P ^c^** | **P_C_ ^d^** | **AR % ^b^** | **No. BRT positive (%)** | **No. NAb negative (%)** | **OR (95% C.I.)** | **P ^c^** | **P_C_ ^d^** | **AR % ^b^** |
| **DQA1*05** | 22 (18.6) | | 39 (36.1) | | 0.41 (0.22-0.74) | 0.0042 | 0.16 | 20.7 | 11 (14.1) | 39 (36.1) | 0.29 (0.14-0.61) | <0.001 | 0.030 | 8.4 |
| **DQB1*02** | 25 (20.5) | | 32 (29.4) | | 0.62 (0.34-1.13) | 0.13 | 1 | 26.1 | 15 (18.5) | 32 (29.4) | 0.55 (0.27-1.1) | 0.092 | 1 | 12.9 |
| **DQB1*03** | 48 (39.3) | | 60 (55.0) | | 0.53 (0.31-0.89) | 0.018 | 0.69 | 26.6 | 35 (43.2) | 60 (55) | 0.62 (0.35-1.11) | 0.14 | 1 | 15.5 |
| **DQB1*06** | 105 (86.1) | | 81 (74.3) | | 2.14 (1.09-4.17) | 0.030 | 1 | 37.0 | 75 (92.6) | 81 (74.3) | 4.32 (1.7-11.02) | 0.0011 | 0.040 | 22.6 |
| **DRB1*01** | 12 (7.1) | | 19 (10.6) | | 0.65 (0.31-1.39) | 0.35 | 1 | 25.5 | N/A | N/A | N/A | N/A | N/A | N/A |
| **DRB1*03** | 25 (15.0) | | 41 (22.8) | | 0.6 (0.35-1.03) | 0.075 | 1 | 25.0 | 14 (12.5) | 41 (22.8) | 0.48 (0.25-0.94) | 0.032 | 1 | 11.4 |
| **DRB1*04** | 41 (24.1) | | 66 (36.7) | | 0.55 (0.35-0.87) | 0.015 | 0.55 | 25.0 | 31 (27.4) | 66 (36.7) | 0.65 (0.39-1.09) | 0.13 | 1 | 14.9 |
| **DRB1*07** | 18 (10.8) | | 24 (13.3) | | 0.79 (0.41-1.51) | 0.51 | 1 | 29.1 | 15 (13.4) | 24 (13.3) | 1.01 (0.5-2.01) | 1 | 1 | 19.0 |
| **DRB1*08** | 28 (16.8) | | 10 (5.6) | | 3.42 (1.61-7.29) | <0.001 | 0.036 | 60.5 | 13 (11.6) | 10 (5.6) | 2.23 (0.94-5.28) | 0.075 | 1 | 32.9 |
| **DRB1*13** | 29 (17.4) | | 39 (21.7) | | 0.76 (0.45-1.3) | 0.34 | 1 | 28.9 | 17 (15.2) | 39 (21.7) | 0.65 (0.35-1.21) | 0.22 | 1 | 14.1 |
| **DRB1*15** | 140 (83.8) | | 100 (55.6) | | 4.15 (2.5-6.88) | <0.001 | <0.001 | 43.3 | 102 (91.1) | 100 (55.6) | 8.16 (4-16.65) | <0.001 | <0.001 | 27.7 |
|  | | **NAb development** | | | | | | | **Biologically relevant titers** | | | | | |
| **IFNβ-1b** | | **No. NAb positive (%)** | **No. NAb negative (%)** | **OR (95% C.I.)** | | **P ^c^** | **P_C_ ^d^** | **AR % ^b^** | **No. BRT positive (%)** | **No. NAb negative (%)** | **OR (95% C.I.)** | **P ^c^** | **P_C_ ^d^** | **AR % ^b^** |
| **Treatment only ^a^** | | n/a | n/a | n/a | | n/a | n/a | 49.3 | n/a | n/a | n/a | n/a | n/a | 15.9 |
| **A*01** | | 36 (27.1) | 16 (28.1) | 0.95 (0.48-1.9) | | 1 | 1 | 48.4 | 23 (37.7) | 16 (28.1) | 1.55 (0.71-3.37) | 0.33 | 1 | 20.2 |
| **A*02** | | 66 (49.6) | 24 (42.1) | 1.35 (0.72-2.53) | | 0.43 | 1 | 53.4 | 32 (52.5) | 24 (42.1) | 1.52 (0.73-3.14) | 0.27 | 1 | 19.0 |
| **A*03** | | 57 (42.9) | 25 (43.9) | 0.96 (0.51-1.8) | | 1 | 1 | 48.8 | 22 (36.1) | 25 (43.9) | 0.72 (0.35-1.51) | 0.45 | 1 | 13.4 |
| **B*07** | | 50 (41.7) | 19 (37.3) | 1.2 (0.61-2.36) | | 0.61 | 1 | 52.1 | 21 (37.5) | 19 (37.3) | 1.01 (0.46-2.21) | 1 | 1 | 16.0 |
| **B*08** | | 20 (16.7) | 10 (19.6) | 0.82 (0.35-1.9) | | 0.66 | 1 | 45.3 | 10 (17.9) | 10 (19.6) | 0.89 (0.34-2.36) | 1 | 1 | 14.7 |
| **B*15** | | 35 (28.9) | 13 (25.5) | 1.19 (0.57-2.5) | | 0.71 | 1 | 52.5 | 19 (33.3) | 13 (25.5) | 1.46 (0.63-3.37) | 0.41 | 1 | 19.8 |

**Table S5. Carrier frequency and absolute risk for HLA allele groups analyzed for association to NAb development and biologically relevant titers within each treatment group (continued).**

|  | **NAb development** | | | | | | **Biologically relevant titers** | | | | | |
| --- | --- | --- | --- | --- | --- | --- | --- | --- | --- | --- | --- | --- |
| **IFNβ-1b** | **No. NAb positive (%)** | **No. NAb negative (%)** | **OR (95% C.I.)** | **P ^c^** | **P_C_ ^d^** | **AR % ^b^** | **No. BRT positive (%)** | **No. NAb negative (%)** | **OR (95% C.I.)** | **P ^c^** | **P_C_ ^d^** | **AR % ^b^** |
| **B*44** | 26 (21.7) | 10 (20) | 1.11 (0.49-2.51) | 1 | 1 | 51.3 | 13 (23.2) | 10 (20) | 1.21 (0.48-3.07) | 0.81 | 1 | 18.0 |
| **C*03** | 38 (31.4) | 19 (35.2) | 0.84 (0.43-1.66) | 0.73 | 1 | 46.5 | 19 (34.5) | 19 (35.2) | 0.97 (0.44-2.14) | 1 | 1 | 15.6 |
| **C*04** | 14 (11.6) | 11 (20.4) | 0.51 (0.22-1.22) | 0.16 | 1 | 35.6 | N/A | N/A | N/A | N/A | N/A | N/A |
| **C*07** | 74 (61.2) | 33 (61.1) | 1 (0.52-1.94) | 1 | 1 | 49.3 | 31 (56.4) | 33 (61.1) | 0.82 (0.38-1.76) | 0.70 | 1 | 14.8 |
| **DQA1*01** | 80 (77.7) | 28 (82.4) | 0.75 (0.28-2.02) | 0.64 | 1 | 47.9 | 35 (74.5) | 28 (82.4) | 0.63 (0.21-1.88) | 0.43 | 1 | 14.6 |
| **DQA1*03** | 47 (45.6) | 12 (35.3) | 1.54 (0.69-3.44) | 0.32 | 1 | 55.7 | 24 (51.1) | 12 (35.3) | 1.91 (0.77-4.74) | 0.18 | 1 | 21.5 |
| **DQA1*05** | 26 (25.2) | 13 (38.2) | 0.55 (0.24-1.24) | 0.19 | 1 | 39.1 | 10 (21.3) | 13 (38.2) | 0.44 (0.16-1.17) | 0.13 | 1 | 9.5 |
| **DQB1*03** | 68 (63) | 21 (60) | 1.13 (0.52-2.48) | 0.84 | 1 | 50.5 | 33 (64.7) | 21 (60) | 1.22 (0.5-2.97) | 0.82 | 1 | 16.9 |
| **DQB1*06** | 72 (66.7) | 25 (71.4) | 0.8 (0.35-1.85) | 0.68 | 1 | 47.6 | 29 (56.9) | 25 (71.4) | 0.53 (0.21-1.32) | 0.18 | 1 | 13.1 |
| **DRB1*03** | 17 (13) | 10 (17.2) | 0.72 (0.31-1.68) | 0.50 | 1 | 42.3 | 10 (16.9) | 10 (17.2) | 0.98 (0.37-2.57) | 1 | 1 | 15.7 |
| **DRB1*04** | 66 (49.3) | 16 (27.6) | 2.55 (1.31-4.97) | 0.0067 | 0.13 | 63.5 | 35 (57.4) | 16 (27.6) | 3.53 (1.64-7.61) | 0.0015 | 0.029 | 28.2 |
| **DRB1*11** | 11 (8.4) | 10 (17.2) | 0.44 (0.18-1.1) | 0.084 | 1 | 32.2 | N/A | N/A | N/A | N/A | N/A | N/A |
| **DRB1*15** | 68 (51.9) | 40 (69) | 0.49 (0.25-0.93) | 0.038 | 0.72 | 42.3 | 25 (42.4) | 40 (69) | 0.33 (0.16-0.71) | 0.0052 | 0.098 | 10.4 |

^a^ The frequency for NAb development and biologically relevant titers for each treatment regardless of genotype.

^b^ Absolute risk for each HLA allele group is calculated with Bayes’ theorem and assessed based on frequency and impact on NAb development within each treatment group.

^c^ Nominal *P*-values from Fishers exact test

^d^ Bonferroni corrected *P*-values (allele groups tested: 12 tests for i.m. IFNβ-1a, 19 tests for IFNβ-1b, 38 tests for s.c. IFNβ-1a)

Abbreviations: AR=absolute risk, C.I.=confidence interval, IFNβ=interferon beta, n/a=not applicable, N/A=not available, NAb=neutralizing antibodies, OR=odds ratio
