# Supplementary material for: Human Leukocyte Antigen Genes and Interferon Beta Preparations Influence Risk of Developing Neutralizing Anti-Drug Antibodies in Multiple Sclerosis
Source: PLoS One. 2014 Mar 7;9(3):e90479. doi: 10.1371/journal.pone.0090479 (PMC3946519; doi:10.1371/journal.pone.0090479)
Supplement: Table S6 — Association of DRB1*04 alleles in all patients and in the IFNβ-1b treated group of patients. (DOC) [file pone.0090479.s006.doc]

**Table S6.** **Association of *DRB1*04* alleles in all patients and in the IFNβ-1b treated group of patients.**

| **All treatments** | **Total** | **NAb positive (%)** | **NAb negative (%)** | **OR ^a^** | **P ^b^** |
| --- | --- | --- | --- | --- | --- |
| DRB1*04 negative (weighted) | 526.1 | 207.4 (66.4) | 318.9 (68.5) | n/a | n/a |
| DRB1*04 subtyped ^c^ | 252 | 105 (33.6) | 147 (31.5) | 1.1 (0.81-1.49) | 0.58 |
| DRB1*04:01 | 167 | 74 (23.7) | 93 (20.0) | 1.25 (0.88-1.76) | 0.21 |
| DRB1*04:02 | 1 | 0 (0.0) | 1 (0.2) | N/A | N/A |
| DRB1*04:03 | 2 | 0 (0.0) | 2 (0.4) | N/A | N/A |
| DRB1*04:04 | 76 | 28 (9.0) | 48 (10.3) | 0.86 (0.53-1.4) | 0.62 |
| DRB1*04:05 | 1 | 1 (0.3) | 0 (0.0) | N/A | N/A |
| DRB1*04:07 | 9 | 4 (1.3) | 5 (1.1) | 1.2 (0.32-4.49) | 1 |
| DRB1*04:08 | 1 | 0 (0.0) | 1 (0.2) | N/A | N/A |
| **IFNβ-1b users** | **Total** | **NAb positive (%)** | **NAb negative (%)** | **OR ^a^** | **P ^b^** |
| DRB1*04 negative (weighted) | 97.9 | 58.7 (50.7) | 42.0 (72.4) | n/a | n/a |
| DRB1*04 subtyped ^c^ | 73 | 57 (49.3) | 16 (27.6) | 2.55 (1.29-5.04) | 0.0089 |
| DRB1*04:01 | 49 | 41 (35.4) | 8 (13.8) | 3.43 (1.48-7.93) | 0.0039 |
| DRB1*04:04 | 24 | 16 (13.8) | 8 (13.8) | 1 (0.4-2.50) | 1 |
| DRB1*04:07 | 2 | 2 (1.7) | 0 (0.0) | N/A | N/A |

^a^ Weighted univariate analysis was done by comparing carriers of a certain *DRB1*04* allele against the *DRB1*04* negatives together with *DRB1*04* positives who were subtyped and who did not carry the allele in question. *DRB1*04* positive individuals who were not subtyped were excluded from analysis (explained in detail in material and methods).

^b^ *P*-values from Fishers exact test.

^c^ Total number of *DRB1*04* subtyped patients. Heterozygous allele carriers occur twice, and homozygous allele carriers occur only once.

Abbreviations: IFNβ=interferon beta, n/a=not applicable, N/A=not available, NAb=neutralizing antibodies, OR=odds ratio.
